# ELEMENT TRIAL: study protocol for a randomized controlled trial on endoscopic ultrasound-guided biliary drainage of first intent with a lumen-apposing metal stent vs. endoscopic retrograde cholangio-pancreatography in the management of malignant distal biliary obstruction

**DOI:** 10.1186/s13063-019-3918-y

**Published:** 2019-12-09

**Authors:** Yen-I Chen, Kashi Callichurn, Avijit Chatterjee, Etienne Desilets, Donnellan Fergal, Nauzer Forbes, Ian Gan, Sana Kenshil, Mouen A. Khashab, Rastislav Kunda, Eric Lam, Gary May, Rachid Mohamed, Jeff Mosko, Sarto C. Paquin, Anand Sahai, Gurpal Sandha, Christopher Teshima, Alan Barkun, Jeffrey Barkun, Ali Bessissow, Kristina Candido, Myriam Martel, Corey Miller, Kevin Waschke, George Zogopoulos, Clarence Wong, for the ELEMENT trial and for the Canadian Endoscopic Research Collaborative (CERC)

**Affiliations:** 1 Division of Gastroenterology and Hepatology, McGill University Health Centre, McGill University, Montreal, QC Canada; 2 Division of Gastroenterology, Centre Hospitalier de l’Université de Montréal, Montreal, QC Canada; 3 Division of Gastroenterology and Hepatology, Ottawa Hospital, University of Ottawa, Ottawa, ON Canada; 4 Division of Gastroenterology, Hôpital Charles-Le Moyne, University of Sherbrooke, Greenfield Park, QC Canada; 5 Division of Gastroenterology and Hepatology, Vancouver General Hospital, University of British Columbia, Vancouver, BC Canada; 6 Division of Gastroenterology and Hepatology, University of Calgary, Calgary, AB Canada; 7 Division of Gastroenterology and Hepatology, Johns Hopkins Hospital, Baltimore, MD USA; 8 Department of Surgery, Department of Gastroenterology and Hepatology, Department of Advanced Interventional Endoscopy, Universitair Ziekenhuis Brussel, Vrije Universiteit Brussel, Brussels, Belgium; 9 Division of Gastroenterology and Hepatology, St-Paul’s Hospital, University of British Columbia, Vancouver, BC Canada; 10 Division of Gastroenterology and Hepatology, St-Michael’s Hospital, University of Toronto, Toronto, ON Canada; 11 Division of Gastroenterology and Hepatology, University of Alberta Hospital, University of Alberta, Edmonton, AB Canada; 12 Department of Surgery, McGill University Health Centre, McGill University, Montreal, QC Canada; 13 Department of Radiology, McGill University Health Centre, McGill University, Montreal, QC Canada; 14 Division of Gastroenterology and Hepatology, Royal Alexandra Hospital, University of Alberta, Edmonton, AB Canada

**Keywords:** Malignant distal obstruction, Endoscopic retrograde, Cholangiopancreatography (ERCP), Endoscopic ultrasound guided-biliary drainage (EUS-BD), Treatment, Trial, Endoscopy

## Abstract

**Background & aims:**

Endoscopic ultrasound guided-biliary drainage (EUS-BD) is a promising alternative to endoscopic retrograde cholangiopancreatography (ERCP); however, its growth has been limited by a lack of multicenter randomized controlled trials (RCT) and dedicated devices. A dedicated EUS-BD lumen- apposing metal stent (LAMS) has recently been developed with the potential to greatly facilitate the technique and safety of the procedure. We aim to compare a first intent approach with EUS-guided choledochoduodenostomy with a dedicated biliary LAMS vs. standard ERCP in the management of malignant distal biliary obstruction.

**Methods:**

The ELEMENT trial is a multicenter single-blinded RCT involving 130 patients in nine Canadian centers. Patients with unresectable, locally advanced, or borderline resectable malignant distal biliary obstruction meeting the inclusion and exclusion criteria will be randomized to EUS-choledochoduodenostomy using a LAMS or ERCP with traditional metal stent insertion in a 1:1 proportion in blocks of four. Patients with hilar obstruction, resectable cancer, or benign disease are excluded. The primary endpoint is the rate of stent dysfunction needing re-intervention. Secondary outcomes include technical and clinical success, interruptions in chemotherapy, rate of surgical resection, time to stent dysfunction, and adverse events.

**Discussion:**

The ELEMENT trial is designed to assess whether EUS-guided choledochoduodenostomy using a dedicated LAMS is superior to conventional ERCP as a first-line endoscopic drainage approach in malignant distal biliary obstruction, which is an important and timely question that has not been addressed using an RCT study design.

**Trial registration:**

Registry name: ClinicalTrials.gov. Registration number: NCT03870386. Date of registration: 03/12/2019.

## Background

EUS-biliary drainage (BD) is a promising alternative to ERCP for the management of malignant distal biliary obstruction. Although ERCP has been the standard of care over the past four decades, the transpapillary route is associated with the risk of post-ERCP pancreatitis (PEP) while the insertion of a metal stent through the tumor can lead to subsequent stent dysfunction secondary to tumor tissue stent ingrowth and/or overgrowth in 20 to 40% of the patients [1–5]. EUS-BD through the transluminal approach is especially adapted to managing malignant distal biliary obstruction since a biliary bypass is created with a stent either through a choledochoduodenostomy (CDS) from the duodenal bulb or a hepatogastrostomy, thereby limiting the risk for PEP and stent tumor tissue ingrowth or overgrowth [6].

Recent small RCTs comparing EUS-BD with ERCP suggest similar technical success and safety profile while potentially being associated with lower rates of stent dysfunction [6–8]. Major limitations of trials having assessed this comparison to date are the small sample sizes with two of the three studies being underpowered in addition to being single center initiatives, thus limiting the generalizability of the findings. In addition, one of the major limiting factors for EUS-BD has been the lack of dedicated devices making the procedure technically complex [9]. Recently, a lumen-apposing metal stent (LAMS) has been developed specifically for EUS-BD that has been approved in Canada, Asia, and Western Europe (not yet FDA approved). Cohort studies have shown encouraging results [10–12]; however, prospective trials, let alone randomized trials, are lacking.

The role of EUS-BD using LAMS as a viable alternative to ERCP as the first-line modality in malignant distal biliary obstruction is a growing question, which has never been studied in a multicenter randomized controlled trial. Our pan-Canadian RCT thus assesses whether EUS-BD with a biliary LAMS is superior to ERCP.

## Methods

### Design and objectives

**Fig. 1:**
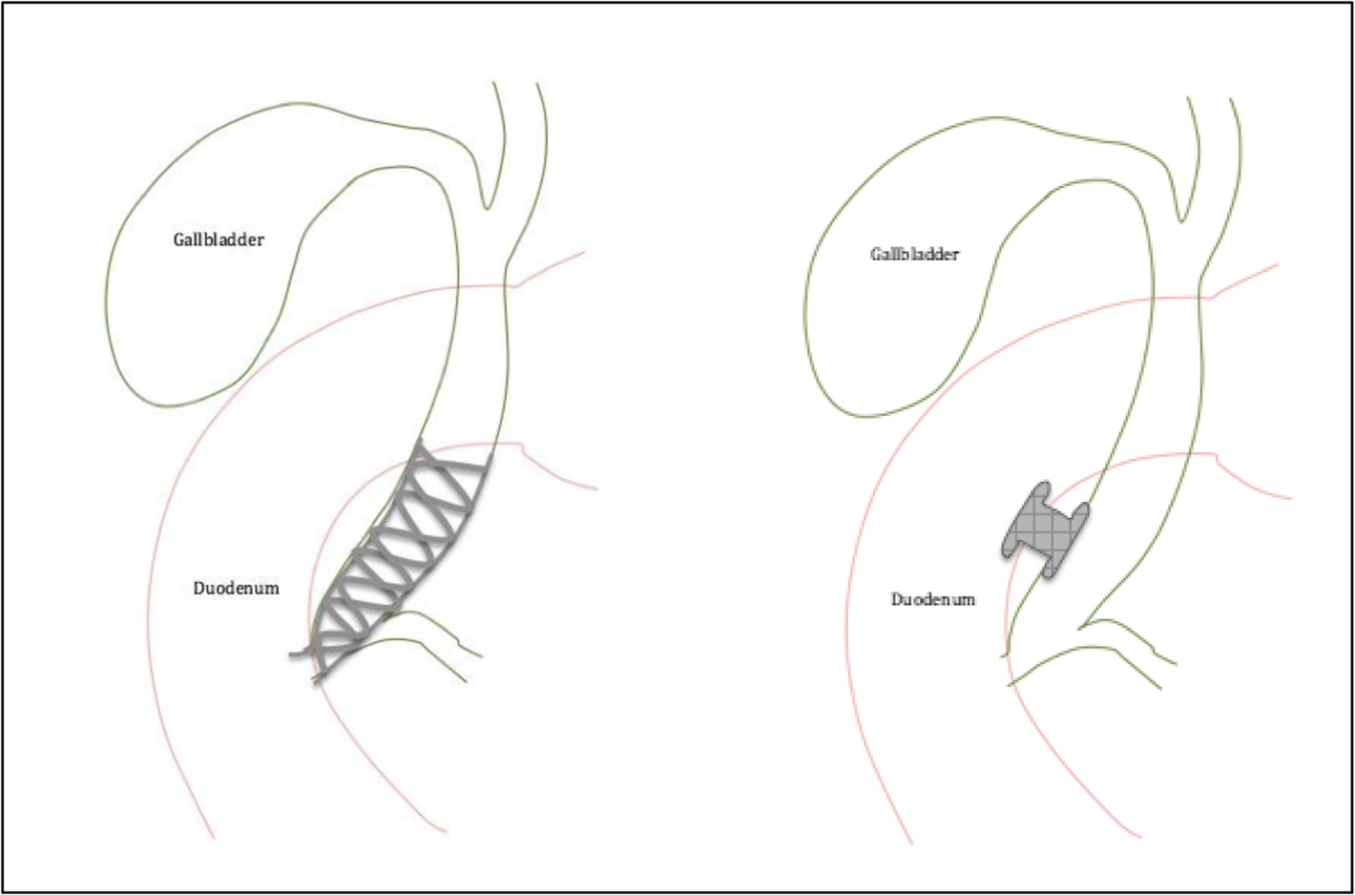
ERCP with traditional metal stent versus EUS-BD with LAMS

The Eus-guided biliary drainage with LumEn apposing Metal stENT vs. ercp (ELEMENT) trial is a multicenter prospective randomized controlled trial, across nine sites in Canada, comparing EUS-CDS with a biliary LAMS vs. ERCP in the initial endoscopic management of malignant distal biliary obstruction (Fig. 1). We hypothesize that EUS-CDS using LAMS is associated with fewer episodes of stent dysfunction and need for re-intervention when compared to standard ERCP while being comparable in safety and technical success in patients with malignant distal biliary obstruction (Additional file 1). At the time of writing this manuscript, this trial has been approved by the IRB of the coordinating center and two additional participating sites. All other sites have submitted their IRB application and will only start patient recruitment once approved.

### Study population

Patients with malignant distal biliary obstruction meeting the criteria below (Table 1) will be considered for consent. All centers involved are busy tertiary care university-affiliated institutions that perform approximately 1000 ERCP and 1000 EUS per year.

**Table 1:** Eligibility criteria

Inclusion criteria (must fulfill all)	
1. Radiological diagnosis (with or without pathological confirmation) of borderline resectable, locally advanced, or unresectable malignant distal biliary obstruction at least 2 cm distal to the hilum (resectability based on tumor staging on axial imaging and physician evaluation)	
2. Elevated liver tests with serum bilirubin at least three times above the upper limit of normal (1.2 mg/dl) Dilated extra-hepatic bile duct measuring at least 1.2 cm on axial imaging or US	
3. Karnofsky index > 30%	
4. ASA score < IV, and	
5. Provision of informed consent	
Exclusion criteria (any of the following)	
1. Hilar obstruction	
2. Uncorrectable coagulopathy and/or thrombocytopenia	
3. Age < 18	
4. Liver metastasis > 30% of the liver volume	
5. Liver cirrhosis with portal hypertension or ascites	
6. Prior biliary sphincterotomy or stent placement	
7. Surgically altered anatomy	
8. Patient with clinical and radiological evidence of gastric outlet obstruction	

### Patient identification and consent

Patients are identified by the treating team through referrals made to the participating physician or participating hospital center for drainage of biliary obstruction. Patients meeting the criteria above are approached by the treating endoscopist prior to the procedure. The endoscopist introduces the trial briefly to the patient to assess whether he or she would be interested in speaking to the research assistant. If agreeable, the patient is then approached by the research assistant who will perform the consent process including a detailed explanation of the trial such as risks, benefits, methods, and inconveniences to the participants. Patients are given a copy of the consent form and given the time to read the entire document and will be given the opportunity to ask any questions prior to consent. The patient is reminded that consent is completely voluntary and can be withdrawn at any time.

### Randomization

**Fig. 2:**
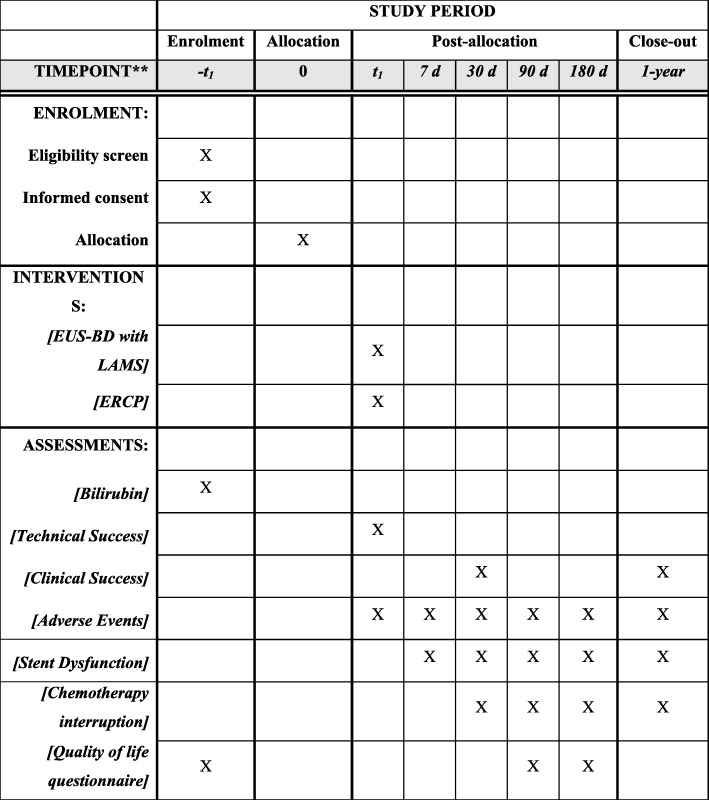
Enrollment, intervention, and assessment in the ELEMENT trial

Patients included are randomized in a 1:1 ratio, in blocks of four, stratified by site and resectability: borderline resectable/locally advanced versus unresectable malignant distal biliary obstruction (Fig. 2 via secure Internet software (randomize.net)). Patients are randomized by a research assistant following consent. The endoscopist is then informed of the treatment allocation and the research nurse will verify that the correct assigned procedure is carried through and documented.

The investigator’s research personnel are responsible for entering all criteria on randomize.net which will then generate the randomization sequence. The research personnel involved in the statistical analysis and patients, but not the endoscopists, are blinded to treatment allocation. To ensure the quality of the trial, unblinding will occur only in exceptional circumstances when knowledge of the treatment allocation is essential for the optimal management of the patient.

### Treatment protocol

Treatment allocation is to EUS-CDS with LAMS vs. insertion of a traditional transpapillary metal stent at ERCP. All procedures are performed by experienced endoscopists with or without trainee involvement. Following informed consent, procedures are either performed under conscious sedation or general anesthesia, as per existing institutional procedural protocols.

#### EUS-BD

A curvilinear endoscope is inserted orally and advanced to the duodenal bulb. Biliary accessibility is then confirmed via EUS from the duodenal bulb and with Doppler to rule out any intervening vessels. In order to maintain stability in the duodenal bulb, the long endoscope position is used when possible. A LAMS (Axios™, Boston Scientific, Marlborough, MA, USA) is then inserted with cautery assistance without tract dilation and deployed under EUS guidance from the duodenal bulb. The choice of stent size is at the discretion of the endoscopist (8 × 8 mm or 6 × 8 mm).

#### ERCP

A duodenoscope is advanced orally to the papilla. The bile duct is then cannulated with a sphincterotome using the guidewire-assisted technique. A cholangiogram is then performed followed by insertion of a self-expanding metal biliary stent. The performance of a biliary sphincterotomy prior to stent insertion and the choice of stent size (10 × 40 mm, 10 × 60 mm, 10 × 80 mm) and stent type (partially covered, fully covered, or uncovered) are recorded and left to the discretion of the endoscopist.

The assigned procedure (EUS-BD or ERCP) will be aborted in case of technical failure including inability to reach the papilla or cannulation failure in case of ERCP and inability to obtain a safe window for LAMS insertion in EUS-BD. A medical effectiveness approach is adopted, allowing additional procedures deemed indicated by the treating team (ERCP, EUS-BD, percutaneous drainage, and/or surgery) following the initial procedure in both groups, which will be recorded.

### Endpoints

The primary endpoint is the rate of stent dysfunction (including both migration and occlusion) needing endoscopic or radiological re-intervention (Table 2). Patients who reach the primary endpoint are considered to have completed the study once hospitalization time for stent dysfunction and delays in chemotherapy treatment have been captured. Patients who do not reach the primary outcome are followed until surgery, death, or up to 1-year post stent insertion.

**Table 2:** Criteria for primary endpoint of stent dysfunction

Meeting one or more of the following criteria:	
□ Suspected cholangitis [13]	
□ Definite cholangitis [13]	
□ 50% increase in bilirubin from the lowest level post index procedure	
□ 20% increase in bilirubin from the lowest level post index procedure AND evidence of obstruction on imaging (US/CT/MRI)	
AND:	
□ Endoscopic or radiological re-intervention confirming stent blockage or migration needing stent cleaning, stent change, and/or additional stent insertion

**Table 3:** Secondary endpoints

1. Technical success defined as successful insertion of a transpapillary stent or choledochoduodenostomy stent at the initial procedure at time of randomization [6–8]	
2. Clinical success defined as 50% decrease in bilirubin < 2 weeks post-stent insertion or less than 25% of pre-procedure bilirubin level within 4 weeks post stent insertion [6]	
3. Stent patency defined as mean time to stent obstruction or migration (patient will be censored at last-follow-up or death)	
4. Proportion of patients with interruptions in chemotherapy treatment due to recurrent stent dysfunction	
5. Early adverse events (within 14 days of index procedure) as defined per the ASGE lexicon for endoscopic adverse events [18], including post-procedural pancreatitis, bleeding, intestinal perforation, and cholangitis	
6. Delayed adverse events (greater than 14 days of index procedure until last follow-up or death)	
7. Cost-effectiveness analysis	
Additional outcomes	
1. Median number of days in delayed chemotherapy treatment if interrupted	
2. Procedure time defined as from insertion of the endoscope to endoscope withdrawal1	
3. Fluoroscopy time	
4. Rate of hospitalization and length of stay	
5. Mortality	

### Data collection

Collected data include primary, secondary (Table 3), and additional endpoints listed above, demographics, ASA score, Karnofsky index, type of sedation used, laboratory investigation including complete blood count, coagulation profile, liver enzymes, tumor staging based on the TNM classification for pancreatic cancer, quality of life data using the EQ-5D, peri-endoscopic data such as use of rectal indomethacin, antibiotics, and technical details including type of endoscope, EUS and ERCP accessories including stent type and size, sphincterotomy or tract dilation performed prior to stent insertion. Data are collected at the index procedure, and also at days 7, 30, 90, 180, 270, and 1 year post-randomization.

All data will be collected by research personnel who have been previously trained to use both the randomization website (randomize.net) and the data capturing software (redCAP). These research personnel are also GCP (Good Clinical Practices) and SOP (Standard Operating Procedures) certified. All data collected will be de-identified, meaning that information such as name, date of birth, hospital identifier, and Medicare number will not be stored in the main database. Each participant will be referred to by their study number. There will be a separate file stored in a password protected and encrypted computer with the decoder of identifiables in case these patients need to be contacted. All data will be stored for 7 years after publication or as requested in Standard Operating Procedure (SOP) from individual sites. Only data relevant to the study as outlined in this protocol will be collected by the research team. All information collected during the research project will remain confidential.

### Follow-up

All patients are followed until either the primary outcome, surgery, death, or a total of 1-year post stent insertion have been reached, whichever comes first. A medical effectiveness approach is adopted, allowing additional therapeutic approaches deemed indicated by the treating team following the initial procedure in both groups, and are recorded.

Follow-up will be conducted over the phone and at subsequent office visits. If the patient is unable to be reached, a letter will be sent to their residence requesting a call back to the research assistant. A chart review can be performed in the event a patient is not able to be reached within a 2-week window of the follow-up date. If follow-up information is unable to be obtained, the patient will be considered a loss to follow-up and their data will documented accordingly. Any patient who deviates from the protocol or withdraws their consent from the ELEMENT trial at any point will be considered a protocol deviation and will be withdrawn from the data monitoring.

### Statistical aspect

#### Sample size calculation

**Fig. 3:**
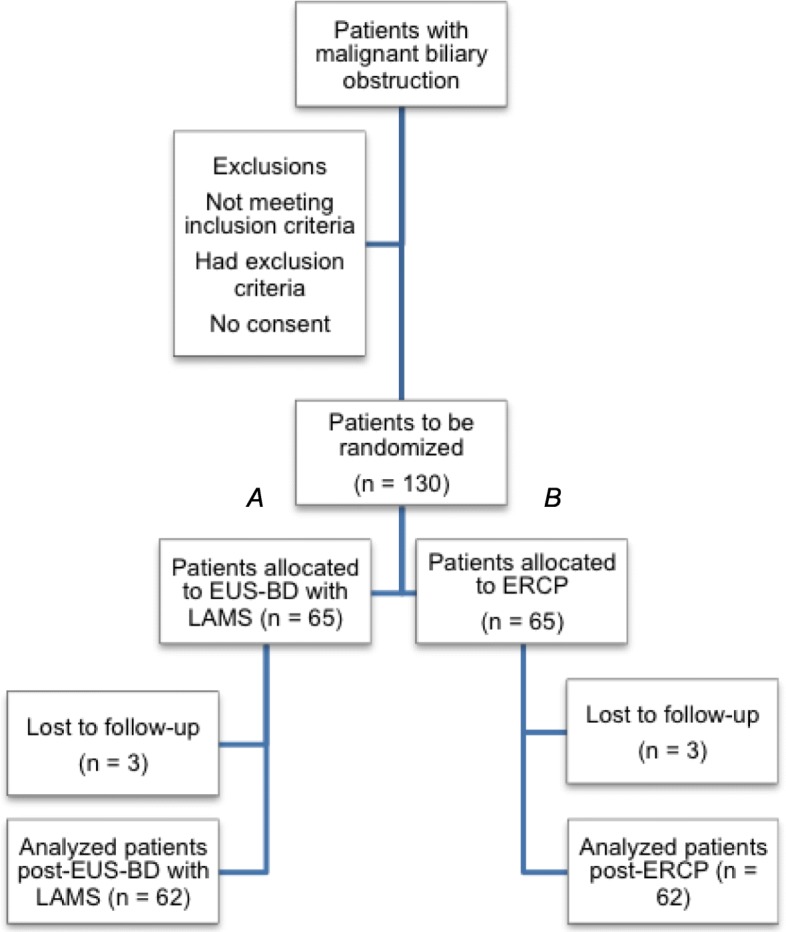
Flowchart ELEMENT trial

The sample size calculation is based on the primary endpoint of rate of re-intervention and we are assessing for superiority. We also include a non-inferiority sample size calculation for the secondary endpoint of technical success to ascertain the adequacy of statistical powering for other outcomes (Fig. 3).

##### Primary endpoint

Rate of re-intervention: Based on RCT data and retrospective series using LAMS [6–8, 10–12], we estimate a re-intervention rate of 30% vs. 10% for the ERCP and EUS-BD groups, respectively up to a 1-year follow-up period. To achieve a statistical power of 80% with a two-sided type I error of 5%, a total of 124 patients (62 in each arm) is required. Taking into account a 5% dropout rate, a final sample of 130 patients (65 patients in each arm) is needed.

##### Secondary endpoints

Technical success: we estimate a technical success of 95% in the ERCP cohort [6–8]. A non-inferiority margin of 10% is implemented. To achieve a statistical power of 80% with a one-sided type I error of 5%, a total of 118 patients (59 in each arm) is required. Taking into account a 5% dropout rate, 124 patients (62 patients in each arm) are needed. Therefore, with a sample size of 130 patients, we are adequately powered for both the primary and main secondary endpoints.

### Analyses

An intention-to-treat analysis along with a secondary per-protocol analysis will be carried out. Descriptive statistics will assess the adequacy of patient characteristics and balancing of the randomization process. Inferential analysis will employ Student’s *t* test and Chi-square testing. Survival analysis using the Kaplan–Meier method will be used to assess stent patency time. A step-wise multivariate regression analysis will be undertaken to identify independent predictors of stent dysfunction and adverse events using a log-rank test. A *p* value of 0.05 or less will be considered statistically significant. All analyses will be performed using SAS version 9.4. No interim analysis is planned given the short accrual time, relative small sample size, and short follow-up period.

#### Cost-effectiveness analysis

A decision tree will be created for the two possible biliary drainage approaches over a 6-month time horizon. Probability assumptions will be extracted from the RCT and the unit of effectiveness will be cost per QALY. Costs will be determined from chart data abstraction of patients in each group to determine resource consumption. The regie de l’assurance maladie du Quebec (RAMQ) and institutional database relating to professional fees, hospitalization (where appropriate), and equipment accounting for direct costs will be utilized. Third-party costs related to the procedure and downstream management costs will be determined based on the RCT resource utilization data. Deterministic and probabilistic sensitivity analyses will be undertaken to assess the robustness of the findings.

#### Trial management

The trial is registered at clinicaltrials.gov (NCT03870386). The McGill University Health Centre (MUHC) is the coordinating center. An experienced trial coordinator (MM) and the principal investigator (YC) will oversee the proposed RCT. Responsibilities will include overseeing all sites’ ethics submissions, REDCAP-based data collection (monitored every 2 weeks), as well as data clean-up, Good Clinical Practice (GCP) adherence, and staff training. MM will also track all sites’ patient accrual, maintain weekly communication with all sites and issue a monthly newsletter on the trial progress. Each study site will require a trained GCP-certified research assistant to ensure timely accrual and complete data collection. The responsibilities of the research assistants include study start-up review study documents, local adaptation of consent form, training, contract, enrolment, data entry, query resolution as well as extra annual administration tasks submissions for continuing REB review, updating investigator site file and essential documents, as well as AE/SAE reporting.

Scheduled site visits will be performed by the primary investigator (YC) and/or the senior trial coordinator (MM). These scheduled audits will occur at each site between 3 and 6 months post-enrolment commencement. Audited activities include informed consent process, inclusion/exclusion criteria documentation, PI and clinical staff responsibilities and qualifications, protocol deviation management, severe adverse event management, and study documentation process.

### Adverse event reporting

#### Definition of adverse event (AE)

An adverse event is the development of an undesirable medical condition or the deterioration of a pre-existing medical condition following or during exposure to a procedure done, whether or not considered causally related to the procedure. An undesirable medical condition can be symptoms (e.g., nausea, chest pain), signs (e.g., tachycardia, enlarged liver), or the abnormal results of an investigation (e.g., laboratory findings, electrocardiogram).

### Definition of Serious Adverse Event (SAE)

A serious adverse event is an AE occurring during the procedure or any time after the procedure, that fulfills one or more of the following criteria:

- Results in death
- Is immediately life-threatening
- Requires in-patient hospitalization or prolongation of existing hospitalization
- Results in persistent or significant disability or incapacity
- Is a congenital abnormality or birth defect
- Is an important medical event that may jeopardize the patient or may require medical intervention to prevent one of the outcomes listed above

#### Coordinating Center

The Coordinating Center is the central location for the collection and maintenance of documentation of adverse events and is responsible for submitting adverse event reports to the REB promptly. The Coordinating Center will maintain documentation of all adverse event reports for each participating site. Adverse event reports submitted to the Coordinating Center must be signed and dated by the participating site’s Principal Investigator. The Coordinating Center will provide appropriate forms to be used by all participating sites for reporting adverse events. Information to be provided must include:

- Subject ID number, and initials
- Date of the event
- Description of the event
- Description of site’s response to the event
- Assessment of the subject’s condition
- Subject’s status on the study (on study, off study, etc.)

#### Participating sites

Participating sites are responsible for reporting adverse events to their REB according to its specific requirements and to the Coordinating Center as follows: Fatal events whether anticipated or unanticipated, and whether or not related to the study, must be reported to the Coordinating Center within 24 h of the participating site Principal Investigator’s learning of the event. Serious and Unanticipated Adverse Events must be reported to the Coordinating Center within 24 h of the participating site Principal Investigator’s learning of the event. Other Serious Adverse Events which may result in a change to the protocol, informed consent, or risk to subjects as specified in the protocol must be reported within three [3] working days of the participating site Principal Investigator’s learning of the event. Adverse events which result in no change to protocol, informed consent, or risk to subjects must be reported to the Coordinating Center on a monthly basis.

All SAEs must be collected whether or not they are considered causally related to the investigational procedure. Investigators and other site personnel are responsible for reporting all casually related SAEs to their REB and the coordinating center.

#### Data Safety Monitoring Board (DSMB)

An independent group of methodologists and physicians will oversee this study DSMB. No member of the DSMB has direct involvement in the conduct of this study or has financial or professional interests that may bias their independent decision making. This committee will meet at the beginning of the trial and will then review possible adverse events on a 6-month basis. Each reviewer will be blinded to the treatment allocation.

During the initial meeting, the committee will discuss the protocol, establish triggers set for data review, and guidelines for monitoring the study. Subsequent meetings will be divided into an open session and closed session. The open session will include the principal investigator, statistician, and all members of the DSMB. The accumulated adverse events, demographic characteristics of enrollees in aggregate, protocol compliance, quality control, and timeliness and completeness of follow-up will be presented by the investigators to the DSMB. The closed sessions will include only members of the DSMB. During this session, members will review the accumulated safety data. The biostatistician for the trial will be available for any questions during this session. Finally, the meeting will conclude with a vote to continue the study, to modify the study, or to terminate the study. A written report by the DSMB will be sent to the principal investigator and REB.

Trial results will be made public through abstract presentation at international gastroenterology conferences including Digestive Disease Week and United European Gastroenterology Week. We will plan to disseminate the results through a full publication in a peer-reviewed medical journal.

## Discussion

The ELEMENT trial is a multi-center randomized trial designed to address the question of whether EUS-BD with LAMS is associated with fewer episodes of stent dysfunction and need for re-intervention compared to standard ERCP in the management of malignant distal biliary obstruction. This clinical trial will also compare the technical and clinical success, adverse events, time to stent dysfunction, and oncological outcomes such as interruptions in chemotherapy, rate of surgical attempt, and survival.

We recently completed a meta-analysis of randomized controlled trials (RCT) comparing EUS-BD vs. ERCP or EUS-BD vs. radiological percutaneous biliary tube drain insertion (PTBD) [14]. Overall, we identified three trials comparing EUS-BD to ERCP [6–8]. Technical and clinical success was similar between modalities; however, rates of re-intervention favored EUS-BD over ERCP (RR 0.40; 95% CI 0.29–094) and PTBD (RR 0.49; 95% CI, 0.28–0.88). In addition, EUS-BD was comparable in terms of safety to ERCP while demonstrating lower rates of adverse events when compared to PTBD (RR 0.59; 95% CI, 0.39–0.87). Although results from this meta-analysis are promising, the studies were single-centered and small, using mostly non-EUS dedicated devices.

The potential for less stent dysfunction requiring reintervention with EUS-BD over ERCP, if shown in our proposed trial, would be of great clinical significance not only because of the diminished morbidity in this often-frail patient population, but also because of the potential impact on oncological outcomes. For patients with metastatic pancreatic cancer with a good performance status, there is a clear survival benefit attributable to FOLFIRINOX therapy [15], which would be necessarily delayed if reinterventions are needed. Furthermore, there has been a push to give neoadjuvant chemotherapy [16, 17] even in resectable disease, meaning that the insertion of a reliable biliary stent that remains patent for longer is likely to become a cornerstone for optimizing oncological outcomes. From a practical efficiency (and perhaps cost-effectiveness) perspective, EUS also has the unique ability to achieve decompression and acquire precise tissue diagnosis at the same clinical setting.

Our trial will be the first multi-center RCT in North America involving the use of a EUS-CDS using a biliary LAMS. The LAMS unique design with bi-flared flanges and short stent length is ideal for apposing two luminal structures for drainage with minimal risk for stent migration. These stents are also cautery-assisted and therefore can be inserted without needing an initial needle puncture and wire insertion, while avoiding the complexity, failure potential, and risks associated with tract dilation and instrument exchange. Retrospective series [10–12] have shown promising results with the largest study demonstrating a technical and clinical success rate of greater than 90%, a low rate of adverse events of 7% with only 9.3% of patients needing re-intervention due to stent dysfunction [12]. Prospective randomized data, however, are lacking and needed. Our multicenter design will not only more definitively provide an adequately powered RCT looking at EUS-BD using a biliary LAMS, but will also produce data generalizable outside the very expert centers.

## Conclusions

The role of EUS-BD in malignant distal biliary obstruction as a primary modality is a timely question that has not been studied with the performance of a multi-center RCT. The ELEMENT trial aims to ascertain whether EUS-CDS with LAMS is superior to ERCP in malignant distal obstruction adopting the primary endpoint of stent dysfunction. The results will likely impact clinical practice in an era of growing neoadjuvant chemotherapy use, by optimizing stable and reliable sustained biliary drainage.

## Trial status

Recruiting, start date: 03/13/2019.

Approximate date when recruitment will be completed: 03/03/2020.

## Supplementary information

**Additional file 1.** SPIRIT 2013 Checklist: Recommended items to address in a clinical trial protocol and related documents*.

## Data Availability

The datasets used and/or analyzed during the current study are available from the corresponding author on reasonable request.

## Abbreviations

BD: Biliary drainage
CI: Confidence interval
ERCP: Endoscopic retrograde cholangiopancreatography
EUS: Endoscopic ultrasound
PTBD: Percutaneous transhepatic biliary drainage
RCT: Randomized controlled trial
RR: Risk ratio

## References

[1] Freeman ML, Nelson DB, Sherman S (1996). Complications of endoscopic biliary sphincterotomy. N Engl J Med.

[2] Kochar B, Akshintala VS, Afghani E (2015). Incidence, severity, and mortality of post-ERCP pancreatitis: a systematic review by using randomized, controlled trials. Gastrointest Endosc.

[3] Almadi MA, Barkun A, Martel M (2016). Self-expandable metal stents versus plastic stents for malignant biliary obstruction. Gastrointest Endosc.

[4] Almadi MA, Barkun A, Martel M (2017). Plastic vs. Self-Expandable Metal Stents for Palliation in Malignant Biliary Obstruction: A Series of Meta-Analyses. Am J Gastroenterol.

[5] Gardner TB, Spangler CC, Byanova KL (2016). Cost-effectiveness and clinical efficacy of biliary stents in patients undergoing neoadjuvant therapy for pancreatic adenocarcinoma in a randomized controlled trial. Gastrointest Endosc.

[6] Paik WH, Lee TH, Park DH (2018). EUS-Guided Biliary Drainage Versus ERCP for the Primary Palliation of Malignant Biliary Obstruction: A Multicenter Randomized Clinical Trial. Am J Gastroenterol.

[7] Bang JY, Navaneethan U, Hasan M (2018). Stent placement by EUS or ERCP for primary biliary decompression in pancreatic cancer: a randomized trial (with videos). Gastrointest Endosc.

[8] Park JK, Woo YS, Noh DH (2018). Efficacy of EUS-guided and ERCP-guided biliary drainage for malignant biliary obstruction: prospective randomized controlled study. Gastrointest Endosc.

[9] Park DoHyun, Yoon WonJae, Choi JunHo, Jang Sunguk, Samarasena Jason, Lee TaeHoon, Paik WooHyun, Oh Dongwook, Song TaeJun, Choi JoonHyuk, Hara Kazuo, Iwashita Takuji, Perez-Miranda Manuel, Lee JohnG, Vazquez-Sequeiros Enrique, Naitoh Itaru, Vila JuanJ, Brugge WilliamR, Takenaka Mamoru, Lee SangSoo, Seo Dong-Wan, Lee SungKoo, Kim Myung-Hwan (2019). The underutilization of EUS-guided biliary drainage: Perception of endoscopists in the East and West. Endoscopic Ultrasound.

[10] Anderloni A, Fugazza A, Troncone E (2019). Single-stage EUS-guided choledochoduodenostomy using a lumen-apposing metal stent for malignant distal biliary obstruction. Gastrointest Endosc.

[11] Jacques Jeremie, Privat Jocelyn, Pinard Fabien, Fumex Fabien, Valats Jean-Christophe, Chaoui Azzedine, Cholet Franck, Godard Bruno, Grandval Philippe, Legros Romain, Kerever Sebastien, Napoleon Bertrand (2018). Endoscopic ultrasound-guided choledochoduodenostomy with electrocautery-enhanced lumen-apposing stents: a retrospective analysis. Endoscopy.

[12] Kunda R, Perez-Miranda M, Will U (2016). EUS-guided choledochoduodenostomy for malignant distal biliary obstruction using a lumen-apposing fully covered metal stent after failed ERCP. Surg Endosc.

[13] Kiriyama S, Kozaka K, Takada T (2018). Tokyo Guidelines 2018: diagnostic criteria and severity grading of acute cholangitis (with videos). J Hepatobiliary Pancreat Sci.

[14] Miller Corey S., Barkun Alan N., Martel Myriam, Chen Yen-I (2019). Endoscopic ultrasound-guided biliary drainage for distal malignant obstruction: a systematic review and meta-analysis of randomized trials. Endoscopy International Open.

[15] Conroy T, Desseigne F, Ychou M (2011). FOLFIRINOX versus gemcitabine for metastatic pancreatic cancer. N Engl J Med.

[16] Conroy T, Hammel P, Hebbar M (2018). FOLFIRINOX or Gemcitabine as Adjuvant Therapy for Pancreatic Cancer. N Engl J Med.

[17] Kindler HL (2018). A Glimmer of Hope for Pancreatic Cancer. N Engl J Med.

[18] Cotton PB, Eisen GM, Aabakken L (2010). A lexicon for endoscopic adverse events: report of an ASGE workshop. Gastrointest Endosc.

